# Ex Vivo Rat Transected Spinal Cord Slices as a Model to Assess Lentiviral Vector Delivery of Neurotrophin-3 and Short Hairpin RNA against NG2

**DOI:** 10.3390/biology9030054

**Published:** 2020-03-15

**Authors:** Azim Patar, Peter Dockery, Siobhan McMahon, Linda Howard

**Affiliations:** 1 Discipline of Anatomy, College of Medicine Nursing and Health Sciences, National University of Ireland Galway, H91 YR71 Galway, Ireland; azimpatar@usm.my (A.P.); peter.dockery@nuigalway.ie (P.D.); 2 Department of Neuroscience, School of Medical Sciences, Universiti Sains Malaysia, Gelugor 11800, Malaysia; 3 Regenerative Medicine Institute (REMEDI), College of Medicine Nursing and Health Sciences, National University of Ireland Galway, H91 YR71 Galway, Ireland

**Keywords:** ex vivo slice culture, organotypic slice culture, lentiviral vector, spinal cord injury, transection injury, neurotrophin-3, short hairpin RNA, NG2 proteoglycan

## Abstract

The failure of the spinal cord to regenerate can be attributed both to a lack of trophic support for regenerating axons and to upregulation of inhibitory factors such as chondroitin sulphate proteoglycans including NG2 following injury. Lentiviral vector-mediated gene therapy is a possible strategy for treating spinal cord injury (SCI). This study investigated the effect of lentiviral vectors expressing Neurotrophin-3 (NT-3) and short-hairpin RNA against NG2 (NG2 sh) to enhance neurite outgrowth in in vitro and ex vivo transection injury models. Conditioned medium from cells transduced with NT-3 or shNG2 lentiviruses caused a significant increase in neurite length of primary dorsal root ganglia neurons compared to the control group in vitro. In an ex vivo organotypic slice culture (OSC) transduction with Lenti-NT-3 promoted axonal growth. Transducing OSCs with a combination of Lenti-NT-3/NG2 sh lead to a further increase in axonal growth but only in injured slices and only within the region adjacent to the site of injury. These findings suggest that the combination of lentiviral NT-3 and NG2 sh reduced NG2 levels and provided a more favourable microenvironment for neuronal regeneration after SCI. This study also shows that OSCs may be a useful platform for studying glial scarring and potential SCI treatments.

## 1. Introduction

The inability of the spinal cord to regenerate after damage makes spinal cord injury (SCI) a devastating condition. Patients can suffer from partial or complete loss of sensation and motor function below the point of injury [1]. SCI represents a clear unmet medical need where new treatments are desperately required, and these need to be evaluated in preclinical studies prior to clinical trials [2]. Animal models of SCI have been developed to better understand the physiology of damage and repair [3]. These animal models are also important for evaluating the efficacy and safety of new therapies to promote regeneration and repair of damaged tissue. However, the animal-based models of SCI require substantial post-operative care and are time consuming and expensive [4]. Furthermore, there is an international effort to replace, reduce and refine animal-based studies (the three Rs). Taken together there is a need for other models of SCI which can be used to evaluate strategies to enhance regeneration and repair.

A promising alternative to in vivo models of SCI is the ex vivo organotypic spinal cord slice culture (OSC) model. This model allows the post-injury interaction of different cells within the spinal cord to be studied, including the interplay of neurons and the glial scar [5]. OSCs are slices that are prepared from the intact rat spinal cord which can be cultured for 2–3 weeks under appropriate conditions [6,7]. The cell populations present in vivo are maintained within OSCs along with three-dimensional connections between neurons, the extracellular matrix and supporting cells. OSCs can reduce some confounding factors found in vivo [7] while reproducing many of the post-traumatic events including some of the acute and secondary injury mechanisms [8,9,10,11,12]. OSC-based work offers the potential of reduced financial cost and faster assessment of therapeutic interventions [7]. OSCs are frequently prepared from neonatal animals, but studies with sections from adult rats showed that the spinal cord morphology and structural integrity was preserved with clear differentiation of white and grey matter [13]. Of course the ex vivo model cannot model the interplay between the immune system and the injury, nor can it be used for the functional/behavioural studies which need to be assessed in vivo.

One of the significant barriers to regeneration of neurons and repair of the spinal cord is the glial scar. This microenvironment, created primarily by reactive astrocytes, includes extracellular matrix (ECM) proteins, in particular chondroitin sulphated proteoglycans (CSPGs) such as brevican and NG2. Studies have shown that ex vivo injured OSCs recapitulate several elements of the glial scar (e.g., upregulation of NG2 and the increase in reactive astrocytes). These suggest that the ex vivo slice culture may be a useful model to assess strategies which modify the glial scar and to determine how these impact spinal cord regeneration and repair.

Lentiviral vectors are an attractive treatment option for central nervous system (CNS) diseases and injuries due to their capacity to transduce both dividing and non-dividing cells [14,15,16,17]. A range of studies have shown the efficient use of lentiviral vectors to overexpress genes of interest (e.g., heat shock protein-27) in stroke [18] and glial cell line-derived neurotrophic factor (GDNF) overexpression in Parkinson’s Disease [19]. Studies in SCI have utilised lentiviral delivery of neurotrophic factors (e.g., NT-3 and brain cell derived neurotrophic factor, BDNF) [20,21] and lentiviral delivery of chondroitinase ABC (ChABC) [22,23] to promote spinal cord repair. Lentiviral vectors encoding NT-3 have been shown to modulate neuronal support over time by promotion of neuronal survival, axonal growth and long distance regeneration [20,24,25,26]. The delayed administration of NT-3 has been shown to improve forelimb function after cervical SCI [27]. The use of NT-3 after SCI has been shown to promote myelination [28,29]. NT-3 has also been successfully administered to injured spinal cord in combination with biomaterials—including collagen, fibrin, Matrigel, hyaluronic acid and chitosan [30,31]. In addition, NT-3 has been used in combination with cell grafts in injured spinal cord [32].

Lentiviral vectors have also been used for targeted short hairpin RNA (shRNA) mediated knockdown of factors after SCI, including glial fibrillary acidic protein (GFAP), vimentin [33,34], NG2 [35,36] and receptor protein tyrosine phosphatase sigma (RPTPσ) and CSPG inhibition [37]. A mouse model of SCI treatment with shRNA against GFAP and vimentin (shGFAP and shVIM) demonstrated improved motor function and increased axonal regrowth and sprouting [34]. Stable expression of shGFAP and shVIM in the lesion site resulted in a significant reduction of astrogliosis. Donnelly et al. (2012) investigated a combinatorial approach using lentiviral vector-mediated shRNA against NG2 (NG2 sh) along with lentiviral-delivery of NT-3 in contused SCI rats. The injured rats showed an improvement in function 2 weeks post-treatment. These behavioural data were supported by the histological observation of increased numbers of neurons and decreased levels of NG2 protein, as well as reduced scar size at the lesion site [35].

NG2 (CSPG4-proteoglycan) is a transmembrane glycoprotein expressed in the intact CNS and upregulated after CNS injury [38,39,40]. NG2 has been reported to be involved in inhibition of axonal regeneration after SCI [40,41,42]. The use of ChABC enzymes to digest the glycosaminoglycan chains of NG2 and related proteoglycans promoted neurite outgrowth and axonal growth [43,44,45,46]. Transduction with a lentiviral vector encoding ChABC has been shown to promote functional recovery in the forelimb post-SCI by reducing chondroitin sulphate chains and enhancing axonal growth after a cervical contusion injury [22].

In this study, we investigated the effect of delivering a combination of Lenti-NT-3 and Lenti shNG2 using an ex vivo model of spinal cord transection injury. We hypothesised that Lenti shNG2 can be used to decrease the CSPG NG2 levels and that a combination of Lenti-NT-3 and Lenti shNG2 creates an improved microenvironment for axonal regrowth and sprouting in this ex vivo model that mimics in vivo studies.

## 2. Materials and Methods

### 2.1. Cell Culture

All cell culture media and reagents were purchased from Sigma-Aldrich (Dublin, Ireland). Human embryonic kidney 293T cells were cultured and maintained in HEK293T cell culture media (88% high glucose Dulbecco’s modified Eagle’s medium (DMEM), 10% foetal bovine serum (FBS), 1% penicillin streptomycin (PS) and 1% non-essential amino acid (NEAA)). Neu7 inhibitory rat astrocytes derived from neonatal rat cortical cultures were a kind gift from Professor James Fawcett, University of Cambridge, UK. The Neu7 cell line expresses a high level of CSPG NG2 protein [47] and is therefore a suitable cell line to investigate knockdown of NG2 in vitro. Neu7 cells were cultured and maintained in Neu7 culture medium (88% low glucose DMEM, 10% FBS, 1% PS and 1% L-Glutamine). All cells were maintained in a cell culture incubator at 37 °C in a 5% humidified CO_2_ atmosphere.

### 2.2. Dorsal Root Ganglia (DRG) Primary Cells

The protocol for harvesting dorsal root ganglia (DRG) neurons was adapted from [36,48]. Briefly, five Sprague Dawley P4 rat pups (Charles River UK Ltd., Margate, UK) at postnatal day (P) 4 were sacrificed by anaesthesia with 5% isoflurane followed by decapitation using a guillotine (Stoelting Co, Germany). DRGs were dissected from the vertebral columns under 16X magnification stereomicroscope (Wild, Switzerland) in sterile Petri dishes (100 mm diameter) Sarstedt (Numbrecht, Germany). Sterile ice-cold Hanks’ balanced salt solution (HBSS) was used to collect the DRG neurons. To inhibit mitosis and promote the survival of neurons, DRGs were cultured in medium containing 88% low glucose MEM, 15% FBS, 1% PS and 3% glucose supplemented with 0.02M 5-Fluro-2′-deoxyuridine, 0.8M uridine and 10ng/mL NGF and 1% L-glutamine at 37 °C in a 5% humidified CO_2_ atmosphere.

### 2.3. NT-3 and pWPT-GFP Lentiviral Vectors

The human NT-3-expressing lentiviral vector was created, produced and validated as described previously [36]. As an easy to visualise control for transduction, the pWPT-GFP lentiviral vector was used; this was a gift from Didier Trono (Addgene plasmid #12255).

### 2.4. shRNA Lentiviral Vectors Targeting NG2 Transcripts

Lentiviral vectors containing shRNAs targeting NG2 (Mission^®^shRNA) were purchased from Sigma-Aldrich (St Louis, MI, USA). In this study, five shRNAs originally designed to target mouse NG2 were tested for their ability to knockdown rat NG2. To select these shRNAs, the mRNA sequence of rat NG2 (NM_031022) was aligned with the sequence of mouse NG2 (NM_139001) using multiple sequence alignment T-coffee tools [49]. shRNAs which targeted regions of the mouse sequence which were identical or very similar to the rat sequence were selected (see Table 1). A vector containing a shRNA which does not target any mammalian transcript (non-targeting control; NTC) was used to control for the effect of transduction and/or selection with puromycin.

### 2.5. Titration of Lentiviral Vectors

Lentiviral vector preparations were titred by performing qPCR of genomic DNA to quantify the increase in *gag* sequences within the target cell genome post-transduction as described by Kutner et al. (2009) [50] and Sastry et al. (2002) [51]. To quantify an absolute number of *gag* sequences within a transduced cell population, *gag* standard curves were generated using plasmid DNA serially diluted to 10^7^ to 10^3^ copies per reaction. The qPCR reactions were performed in 10 µL total volume PCR reactions in triplicate using a primers pair specific for the *gag* sequence (GGAGCT AGACGATTCGCAGTTA and GGTTGTAGCTGTCCCAGTATTTGTC). The qPCR reactions were run on a StepOnePlus™ real time PCR machine using SYBR Green Master Mix (Thermo Fisher Scientific, Loughborough, UK). The cycling conditions were as follows: 95 °C for 15 min (polymerase activation) followed by 40 cycles of amplification (95 °C denaturation for 15 s, 55 °C annealing for 30 s, 72 °C elongation for 30 s) and a final extension at 72 °C for 30 s. To determine the total number of integrations in the wells of cells, the gag per genome value was multiplied by the number of cells seeded. In this study, we seeded 1 × 10^5^ cells in one well of a six-well plate. To determine the titre in transducing units (TU/mL), the number of integrations per well of cells was divided by the volume of the vector preparation used to transduce the cells.

### 2.6. NT-3 ELISA

To ascertain the amount of NT-3 secreted by Lenti-NT-3-transduced cells, a DuoSet^®^ELISA Human NT-3 Kit was used according to the manufacturer’s protocol with some slight modification. Serial dilutions of standards, samples and detection antibody (200 ng/mL) were freshly prepared in 1% BSA in 1× PBS standards, and samples (50 µL volume) were added to the relevant wells. The plate was covered and incubated for 2 h at room temperature. The 96-well plate was washed three times with 400 µL wash buffer and blotted using clean towel paper. The streptavidin-HRP (1:200 dilution) was added into the well and incubated for 20 min. The 96-well plate was washed three times with 400 µL wash buffer and blotted with clean towel paper. The substrate solution was added and incubated for another 20 min. After 20 min of incubation, 50 µL of 2 N Sulphuric acid (H_2_SO_4_) stop solution was added to the well to halt the reaction. The absorbance was read at 540 to 570 nm wavelength using a Wallac Victor™ 3R Plate Reader (Perkin Elmer, Shelton, CT, USA). Each experiment was run in triplicate.

NT-3 ELISA was also used to determine the level of NT-3 protein in media isolated from spinal cord slices transduced with NT-3 lentiviral vector and combination lentiviral vector NT-3/NG2 sh1 vector. The media were harvested from transduced slices at days 3 and 7 post-transduction.

### 2.7. DRG Neurite Outgrowth Assay

DRG neurons were harvested as outlined above. Prior to DRG cell seeding, eight-well chamber slides (Ibidi GmbH, Martinsried, Germany) were coated with 5 µg/cm^2^ of bovine collagen type I (Corning BD Biosciences, USA) for one hour at room temperature to promote attachment of DRG neurons. A total of 1000 DRGs were seeded into each collagen-coated well and incubated with 200 µL DRG culture medium overnight.

To determine whether NT-3 produced by Lenti-NT-3-transduced cells was functional, 200 µL medium from 293T cells transduced with 1 × 10^8^ TU/mL or 5 × 10^8^ TU/mL of Lenti-NT-3 vector was used to culture DRG neurons for 3 days in vitro. As controls, DRGs were treated with 200 µL 293T medium from untransduced cells or with DRG culture medium.

To analyse the effect of shNG2 lentiviral vectors, 200 µL of medium from Neu7 cells transduced with shNG2 lentiviral vector (NG2 sh1, sh2, sh3, sh4 or sh5) was isolated and incubated with DRG neurons for 3 days in vitro. As controls, DRGs were treated with 200 µL Neu7 cell medium from untransduced cells, from cells transduced with the non-targeting control vector and with DRG culture medium. Each experiment was carried out in triplicate.

### 2.8. Immunocytochemical Staining

#### 2.8.1. DRG Immunocytochemical Staining

DRG neurons were fixed with 4% paraformaldehyde (PFA) for 15 min then washed in 1× PBS three times. To block non-specific staining, the DRG neurons were incubated with 10% normal goat serum (NGS) in 1× PBS with 0.5% Tween-20 for 2 h at room temperature. The blocking solution was aspirated from the wells and replaced with βIII-tubulin (Cell Signalling Tech, Beverly MA, USA) primary antibody diluted (1:400) in 1× PBS overnight at 4 °C. DRGs were washed with 1× PBS three times and were incubated with goat anti-rabbit secondary antibody (1:200) Alexa 594 (Invitrogen, Loughborough, UK) for 2 h at room temperature. The DRGs were washed three times with 1× PBS. To counterstain the nuclei, the cells were incubated with 1µg/mL DAPI (Thermo Fisher Scientific) for 5 min at room temperature. The DRGs were washed with 1× PBS three times and were stored in 1× PBS prior to confocal imaging. Projected confocal image stacks of the immunostained DRGs were used in the stereological analysis of neurite outgrowth. Random fields of view (n = 6) were captured from each sample. ImageJ software was used to split the two channels (red immunostained DRGs and blue DAPI stained nuclei), and these were measured separately using the de-interleave ImageJ editing tool. The DRG images were inverted to greyscale. A square grid was generated using an ImageJ built-in macro, and this grid was placed on the top of the DRG images. To measure the total length per unit area of neurite outgrowth, the number of βIII-tubulin positive neurites crossing a line on the grid was counted (I). The distance between each line on the grid was measured (T). The total length of neurite outgrowth per unit area was estimated using the formula described by Donnelly et al. [36]: total length/unit area = π/2 × I/2 x T. The results are presented as mean ± SEM. The mean differences of DRG neurite length was analysed using one-way ANOVA between conditioned media from non-targeting control; DRG media only; conditioned media from NG2 sh1, sh2, sh3, sh4 and sh5 and transduced cell media followed by Tukey’s post-hoc test.

#### 2.8.2. Neu7 Cell Immunocytochemical Staining

Immunocytochemistry was used to assess NG2 levels in Neu7 cells. Neu7 cells were seeded into eight-well chamber slides at 2000 cells per well in Neu7 cell culture medium and incubated overnight at 37 °C and 5% CO_2_/90% humidity. Medium was removed, cells were washed with 1× PBS, fixed with 4% PFA and nonspecific staining was blocked as above. An antibody against NG2 (AB5320, 1:200; Merck Millipore, Darmstadt, Germany), GFAP (Z0334; 1:200; DAKO, Glostrup, Denmark) or CS-56 (C8035; 1:200; Sigma-Aldrich ((Dublin, Ireland) was added. After 24 h of primary antibody incubation, the Neu7 cells were washed with 1× PBS three times (5 min/wash). The Neu7 cells were then incubated with secondary antibodies for 2 h at room temperature, washed and cell nuclei stained as above. Cells were imaged using confocal microscopy.

### 2.9. shNG2 Lentiviral Vector Transduction of Neu7 Cells

The efficiency of the shRNA NG2 knockdown was evaluated by measuring the decrease in NG2 protein levels produced by Neu7 cells. The five shNG2 vectors (NG2 sh1, 2, 3, 4 and 5) were used to transduce Neu7 cells. A non-targeting shRNA acted as a control for transduction and puromycin selection. A TurboGFP™ lentiviral vector was used to visualise transduced cells. Three days post-transduction, untransduced cells were killed by adding puromycin (4 µg/mL) to the culture medium until a control well of untransduced cells had all died. NG2 protein expression levels were evaluated using Western blot analysis and immunocytochemistry for NG2.

### 2.10. NG2 Western Blot

Cell extracts from Lenti-NG2 shRNA transduced and control cells (15 µg of protein in 30 µL of lysis buffer) were boiled at 95 °C for 5 min and separated on an 8% SDS PAGE gel then wet transferred to a PVDF membrane overnight at 90 mA on ice. Non-specific protein binding was blocked in 5% non-fat dry powdered milk in tris-buffered saline (TBS) and 0.1% Tween-20 (TBST) for 1 h at room temperature on a shaker at 90 rpm. After blocking, the membrane was washed in TBST three times then incubated in NG2 primary antibody (1:200) (Cell Signaling Technology, Beverly MA, USA) in 3% non-fat powdered milk in TBST overnight at 4 °C. The membrane was washed in TBST three times (5 min per wash) then incubated with an anti-rabbit secondary-HRP (1:10,000) in 3% non-fat powdered milk in TBST for 90 min on the shaker. The membrane was then washed in TBST three times (5 min per wash). The membrane was incubated with SuperSignal™ West Pico Chemiluminescent Substrate for 3 min prior to imaging. The blot was imaged using a LICOR Odyssey Imager (LICOR, Nebraska, USA).

### 2.11. Measurement of Corrected Total Cell Fluorescence (CTCF) of NG2 in Transduced Neu7 cells

The CTCF was determined for untransduced and transduced Neu7 cells immunocytochemically stained for NG2. In ImageJ a standard oval shape was drawn (153.96 mm^2^) and placed randomly on the top of each image. The fluorescence intensity was measured by measuring the area, integrated density and mean grey values of the area occupied by the box. In each image, eight random GFP-expressing cells (i.e., cells transduced with the shRNA vector) were selected and the mean intensity of NG2 protein signal was calculated from each image. The exposure time and gain were kept the same in each channel—GFP exposure/gain: 500/50, NG2 exposure/gain: 500/80 and DAPI exposure/gain: 500/80.

### 2.12. Animals

Sprague Dawley rats (Charles River UK Ltd., Margate, UK) were used in this study. All housing and surgical procedures carried out in this study were approved by the Animal Care Research Ethics Committee (ACREC) at the National University of Ireland, Galway. Postnatal day (P) 4 rat pups were sacrificed by anaesthesia with 5% isoflurane followed by decapitation using a guillotine (Stoelting Co, Germany). The bodies were kept in sterile dishes on ice prior to spinal cord harvesting. Spinal cord isolation was performed in a class II biological safety cabinet under aseptic conditions. The skin was incised using a sterile blade #10 (Swann-Morton, Sheffield, UK) along the midline of the dorsum. A small transverse incision was made on the sacral vertebrae. The spinal cords were flushed from the vertebral column using a 1ml syringe fitted with an 18G needle and filled with 1× ice-cold phosphate-buffered saline (PBS; pH 7.4) [52]. The spinal cords were suspended in ice-cold artificial cerebrospinal fluid (aCSF; pH 7.4) containing 126mM NaCl, 2.5mM KCl, 1.25mM NaH_2_PO_4_.H_2_O, 2mM CaCl_2_.2H_2_O, 2mM MgSO_4_.7H_2_O and 10mM glucose. The meninges were gently dissected away using sterile fine forceps in ice-cold sucrose aCSF using a stereomicroscope (Wild MZ32, Jena, Germany).

### 2.13. Spinal Cord Transection and Lentiviral Vector Transduction

Spinal cords were isolated from three litters of P4 pups with (12 pups per litter). From one litter, four spinal cords were placed into three separate Petri dishes. Each spinal cord was cut into pieces 1cm in length and transferred to sterile tissue chopper discs. Longitudinal sections of spinal cord were prepared and cultured as described previously [5,53]. In brief, spinal cord sections were cut to 350µm thickness on the McIIwain tissue chopper (Mickle Laboratory Engineering, Surrey, UK). The spinal cord tissue slices were transferred to 30 mm diameter Millicell^®^ cell culture inserts (Catalogue number PICM0RG50: Merck Millipore, Darmstadt, Germany). The slices were selected from the middle of the spinal cord and had a good proportion of white and grey matter distribution. Two spinal cord slices were cultured on each insert in six-well trays containing 1 mL incubation media: 48% MEM, 25 mM Hepes, 25% heat-inactivated horse serum, 2 mM glutamine, 1% PS, 1% N-Acetyl L-Cysteine and 25% HBSS. Slices were maintained in spinal cord slice culture medium at 37 °C in a 5% humidified CO_2_ atmosphere. After 4 days in culture, the tissue slices were separated into: (1) control/uninjured group and (2) injured group. A transection injury was performed on the spinal cord slices within injury group midway along the length of the tissue slices using two sterile scalpel blades #10 (Swann-Morton, Sheffield, UK) attached to a scalpel handle arranged 460 µm distance apart. The injury created was complete through the full thickness from the top to bottom of each slice. The injury gap area decreased from days 3 and 10; however, the area of gap between the two sides was still visible after 10 days in culture. Medium was changed every two days.

### 2.14. NT-3 and shNG2 Lentiviral Vector Transduction of Spinal Cord Slices

To examine the effect of NT-3 lentiviral vector and combination lentiviral NT-3/sh1 NG2 knockdown on ex vivo spinal cord slices, the control and injured slices were either untransduced or else transduced with the following lentiviral vectors: non-targeting control, GFP, NT-3 and a combination of NT-3 with NG2 sh1. For transduction, on day 3, 166 TU of vector (GFP, non-targeting control, or sh1NG2) was pipetted onto each slice. The slices in the NT-3 group and combination NT-3/NG2 sh1 were transduced with 25 × 10^5^ TU NT-3 lentiviral vector. The spinal cord slices were cultured for 7 days after lentiviral transduction.

### 2.15. Tissue Fixation and Immunohistochemistry

On day 7 post-transduction, medium was removed from each well and replaced with 1× PBS. The spinal cord slices were fixed with 4% PFA for 24 h at 4 °C. These slices were immunohistochemically stained for βIII-tubulin, a marker of neurons or for the CSPG NG2. The spinal cord tissue slices were washed in 1× PBS three times (5 min per wash). The spinal cord tissue on the inserts was transferred to 24-well trays by cutting the membrane of the inserts into smaller pieces to fit inside the 24-well tray wells. Immunohistochemical staining was performed on the tissue slices in the 24-well trays. To block nonspecific staining, the spinal cord slices were incubated with 10% NGS in 1× PBS with 0.5% Tween-20 for 2 h at room temperature. The blocking solution was removed and replaced with primary antibody solution diluted in 1× PBS blocking buffer overnight at 4 °C. Primary antibodies used in this study were mouse anti-βIII-tubulin (Merck Millipore, Germany; 1:100) and mouse anti-NG2 (Merck Millipore, Darmstadt, Germany; 1:200). After a 24 h primary antibody incubation, the slices were washed with 1× PBS three times (5 min per wash), then incubated with secondary antibodies for 2 h at room temperature. The secondary antibodies used in this study were goat anti-rabbit Alexa Flour 594 (Invitrogen, Loughborough, UK; 1:200) and goat anti-mouse Alexa Flour 488 (Invitrogen, Loughborough, UK, UK; 1:200). The slices were washed with 1× PBS three times (5 min per wash). To counter stain the cell nuclei, the slices were incubated with 1 µg/mL DAPI (Thermo Fisher Scientific) for 15 min at room temperature. The slices were washed with 1× PBS three times (5 min per wash) and were left in 1× PBS at 4 °C until ready for imaging. A negative control was carried out where the primary antibody was replaced with buffer.

### 2.16. Imaging

Confocal images of DRGs and Neu7 cells on eight-well chamber slides were captured using a 20X lens on an Andor spinning disc confocal microscope (Andor Technology Ltd., Bristol, UK). To examine the immunopositive cells, confocal z-stack images were captured at 1 µm z step distance apart. Spinal cord slices were also imaged using the Andor spinning disc confocal microscope at 10×. Projected confocal image stacks were captured of immunohistochemically stained spinal cord slices. All imaging was carried out using the same exposure time and emission gain for all spinal slices.

### 2.17. Stereology

Projected confocal images of control and injured spinal cord slices 7 days post-transduction were examined using stereology to determine the volume fraction (Vv) of βIII-tubulin and Vv NG2 transduced cells in the spinal cord slices. ImageJ software was used to generate a square grid. The Vv of the immunohistochemical staining was estimated by dividing number of intersection points on the grid resting on immunostained cells by the total number of intersection points on the grid resting on tissue.

Following culture, staining and imaging, the injured spinal cord slices were divided into three regions of interest: injury zone (IZ) i.e., the area of the lesion site; the scar zone (SZ) defined as a distance of 100 µm from the edge of the lesion site and the near scar zone (NSZ) defined as a 100 µm distance from SZ. The cuts through the slices leave blade marks on the inserts used to culture the slices which can easily be visualised through the microscope; these defined the injury zone. The Vv immunohistochemical staining was determined in each zone of interest.

### 2.18. Statistics

All data collected from the stereological analysis were saved in Microsoft Excel 2013 (Microsoft Office, USA). Statistical analysis was carried out using Minitab software (Minitab Incorporation, USA). All the results were illustrated using Graphpad Prism software (Prism 7, San Diego, CA, USA). To test for differences between the parameters examined in all molecular and in vitro data, a one-way analysis of variance (ANOVA) was performed followed by post-hoc Tukey’s test. For ex vivo slice culture analysis, a two-way ANOVA was carried out, followed by Tukey’s multiple comparison test. Statistical significance was set at probability (*p*) value less than 0.05, 0.01 and 0.001. All the results are presented as mean ± standard error of the mean (SEM).

## 3. Results

### 3.1. Selection of shNG2 Lentiviral Vectors

The aim of this work was to determine the effect of Lenti-NT-3, either alone or in combination with Lenti-NG2 shRNA, on the microenvironment of the lesioned spinal cord using organotypic spinal cord slice cultures. Lentiviral vectors encoding shRNAs targeting the NG2 transcript of *Mus musculus* but not *Rattus norvegicus* were commercially available at the initiation of this study. Rat and mouse NG2 coding sequences were aligned, and two mouse shRNAs were identified which targeted sequences identical between the rat and mouse transcripts (NG2 sh1 and sh2, see Table 1). Three mouse NG2 shRNAs were identified which targeted a sequence with one mismatched nucleotide between the rat and mouse transcripts (sh3, sh4 and sh5; see Table 1). The lentiviral vectors used in this study are illustrated in Figure S1. As a control for transduction, selection and shRNA production, a non-targeting control (NTC) vector was used. This contains a shRNA insert that does not target any known mammalian transcript.

### 3.2. Cells Transduced with NT-3 Lentiviral Vector Produce Significant Levels of NT-3 Protein Which Is Able to Promote DRG Neurite Outgrowth

**Figure 1:**
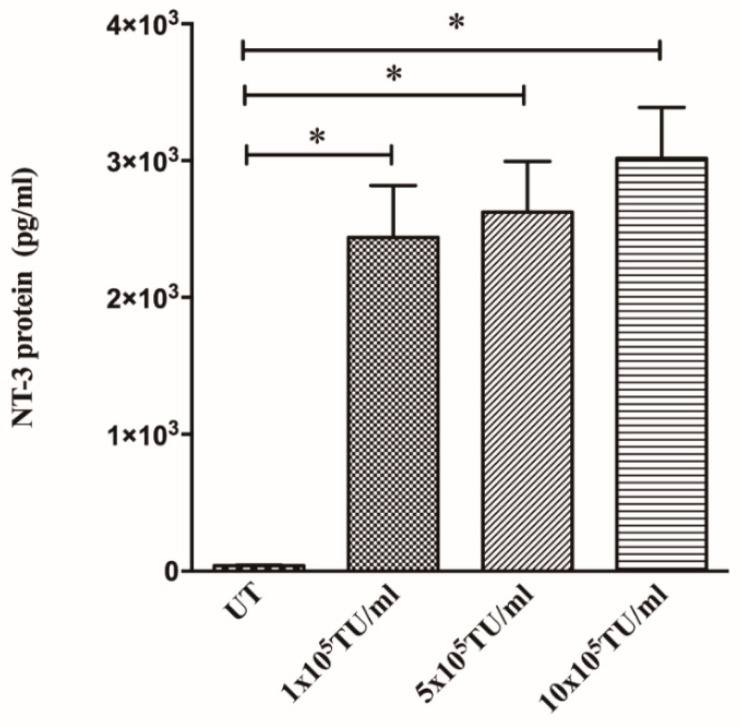
293T cells produce significantly more NT-3 protein following transduction with NT-3 lentiviral vector. Graph shows the concentration of NT-3 protein, determined by ELISA, in media from untransduced (UT), 1 × 10^5^ TU/mL, 5 × 10^5^ TU/mL and 10 × 10^5^ TU/mL. Lenti-NT-3-transduced 293T cells after 3 days in culture. Mean ± SEM. * *p* ≤ 0.05, ** *p* ≤ 0.01, *** *p* ≤ 0.001. n = 3. Tukey’s post-hoc test using one-way ANOVA.

**Figure 2:**
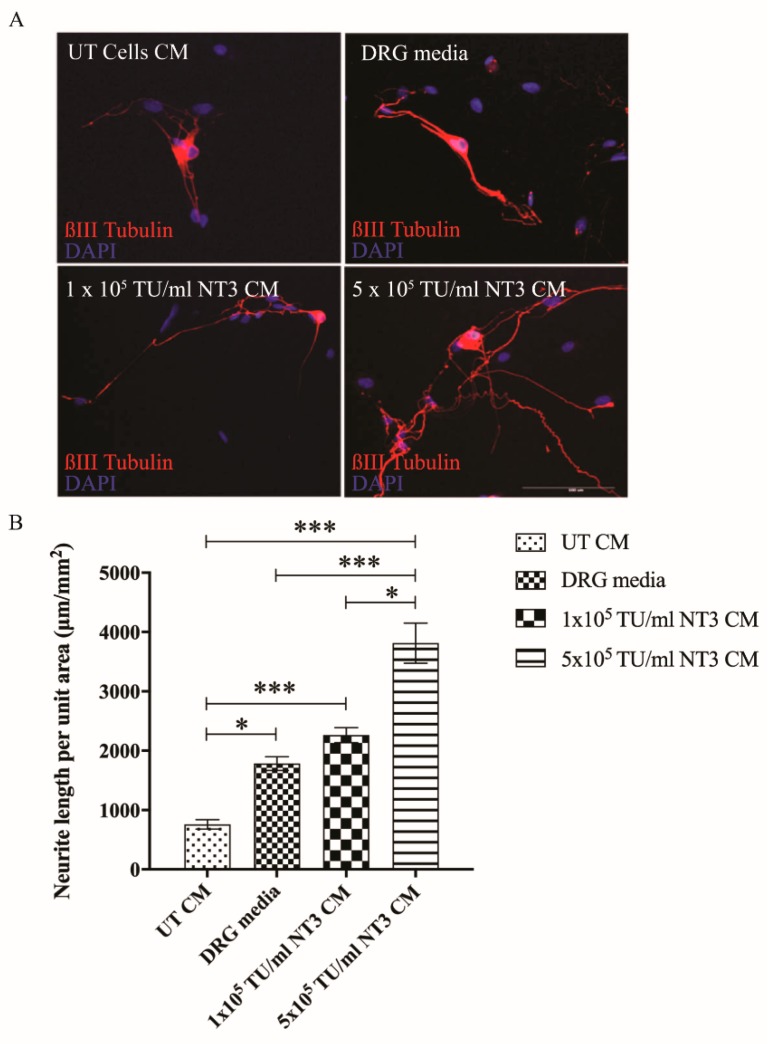
NT-3 produced from 293T transduced cells promotes neurite outgrowth in vitro. Photomicrographs show representative images of projected confocal images of dorsal root ganglia (DRG) neurons after treatment with media from untransduced cells (UT), DRG media (positive control), media from 293T cells transduced with 1 × 10^5^ TU/mL or 5 × 10^5^ TU/mL of NT-3 lentiviral vector (**A**). Scale bar = 200 μm. Graph shows the neurite length per unit area measured using stereology. Significance compared to untransduced cell media is shown (**B**). Data shown as mean neurite length per unit area ± SEM. * *p* ≤ 0.05, ** *p* ≤ 0.01, *** *p* ≤ 0.001. n = 3. Tukey’s post-hoc test using one-way ANOVA.

The 293T cells transduced with Lenti-NT-3 at 1 × 10^5^, 5 × 10^5^ or 10 × 10^5^ TU/mL all produced significantly more NT-3 than untransduced cells demonstrated by ELISA validating the function of the vector (Figure 1). The levels of NT-3 produced by transduced cells (2438 pg/mL) are of biological relevance, as results obtained previously in our laboratory indicated that recombinant NT-3 protein at 100 pg/mL concentration (or higher) caused a significant increase in DRG neurite length [36]. The functional effect of NT-3 protein produced by Lenti-NT-3-transduced 293T cells (1 × 10^5^ TU/mL or 5 x 10^5^ TU/mL) was confirmed when conditioned media from these cells caused a significant increase in DRG neurite length compared to untransduced 293T cell media (Figure 2). These studies demonstrated that cells transduced with NT-3 lentiviral vector secreted functional NT-3 protein capable of promoting DRG neurite outgrowth. Interestingly, conditioned medium from 293T cells transduced with 5 × 10^5^ TU/mL vector lead to a significantly greater increase in DRG length than medium from cells transduced with 1 × 10^5^ TU/mL, even though both conditioned media contained similar levels of NT-3. The reasons for this difference are not yet understood.

### 3.3. Transduction with Lentiviral shNG2 Reduced NG2 Protein Neu7 Cells

**Figure 3:**
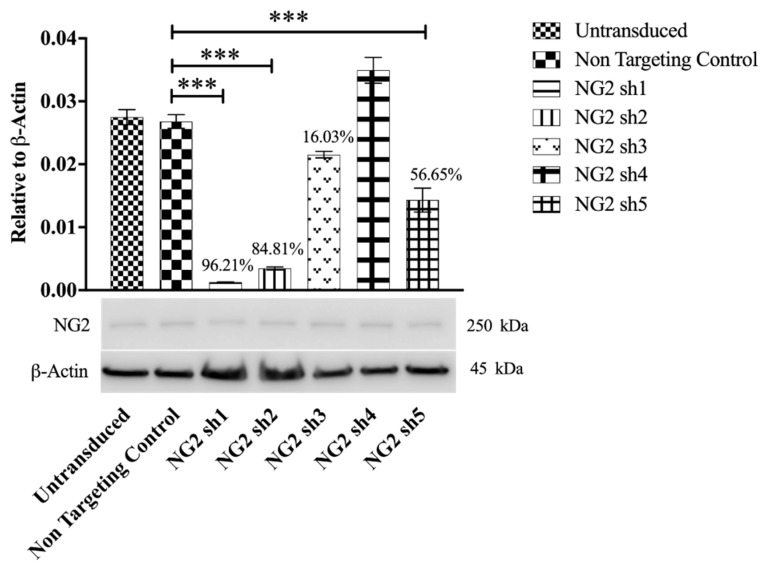
NG2 sh1 and NG2 sh2 lentiviral vectors significantly reduce NG2 protein in Neu7 cells. Graph shows Western-blot-based quantitation to ascertain the knockdown efficacy of non-targeting control, NG2 sh1, NG2 sh2, NG2 sh3, NG2 sh4 and NG2 sh5 in Neu7 cell at the protein level. All the samples were normalised to β-actin. The statistical significance is presented compared to cells treated with non-targeting control. * *p* ≤ 0.05,** *p* ≤ 0.01, *** *p* ≤ 0.001. n = 3. Tukey’s post-hoc test using one-way ANOVA.

The astrocytic cell line Neu7, which expresses NG2, was used to determine the ability of lentiviral shRNAs to knockdown rat NG2 protein [54,55,56]. Neu7 cells were transduced with each lentiviral vector encoding NG2 shRNA (sh1, sh2, sh3, sh4 or sh5). As a control, cells were transduced with a vector encoding a non-targeting shRNA. The puromycin resistance gene on the lentiviral vector allowed selection of transduced cells. Three of the tested shRNAs (NG2 sh1, NG2 sh2 and NG2 sh5) significantly reduced the level of NG2 protein compared to the non-targeting shRNA control and to untransduced cells as determined using Western blot analysis (Figure 3, Figure S2 and Table S1). NG2 sh1 treated cells showed the greatest decrease in NG2 protein level (96%) compared to non-targeting control treated cells.

**Figure 4:**
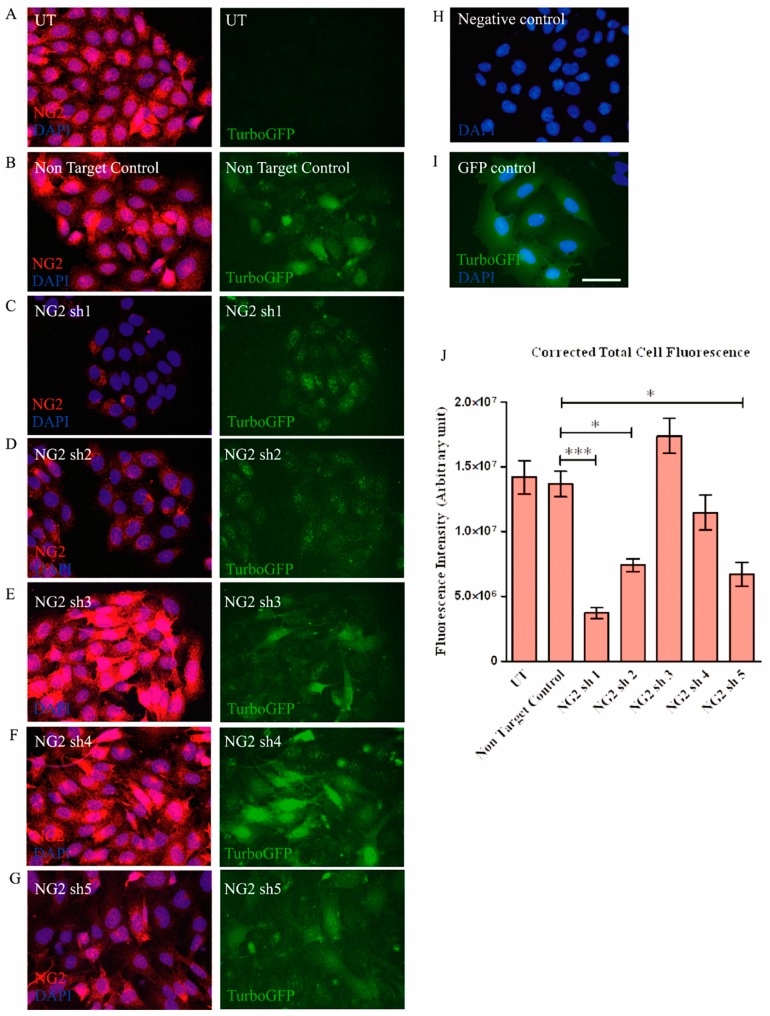
NG2 sh1, NG2 sh2 and NG2 sh5 lentiviral vectors significantly reduce NG2 protein in Neu7 cells in vitro. Photomicrographs show representative projected confocal images of corrected total fluorescence of untransduced control (**A**), non-targeting control (**B**), NG2 sh1 (**C**), NG2 sh2 (**D**), NG2 sh3 (**E**), NG2 sh4 (**F**), NG2 sh5 (**G**), negative control (no primary antibody) (**H**) and GFP control (**I**) after 10 days of puromycin selection. Graph shows the corrected total cell fluorescence of NG2 protein (**J**). Scale bar = 50 μm. Mean ± SEM. * *p* ≤ 0.05, ** *p* ≤ 0.01, *** *p* ≤ 0.001. n = 3. Tukey’s post-hoc test using one-way ANOVA. Significance is reported in comparison to the cells transduced with the non-targeting control.

Immunocytochemistry was performed to confirm the reduction in NG2 protein in Neu7 cells via measurement of total cell fluorescence intensity (Figure 4). In this experiment, the NG2 sh1, NG2 sh2 and NG2 sh5 lentiviral vectors caused a significant reduction of fluorescence intensity of NG2 protein in Neu7 cells compared to the non-targeting shRNA control group. Taken together, these results indicate that transduction with NG2 sh1, NG2 sh2 and NG2 sh5 lentiviral vector causes a significant reduction in NG2 protein in the astrocytic cell line Neu7 in vitro. Furthermore NG2 sh1 was able to significantly reduce NG2 protein production within 3 days of transducing Neu7 cells (Figure S3).

### 3.4. Lentiviral shNG2 Knockdown of NG2 Promotes DRG Neurite Outgrowth In Vitro

**Figure 5:**
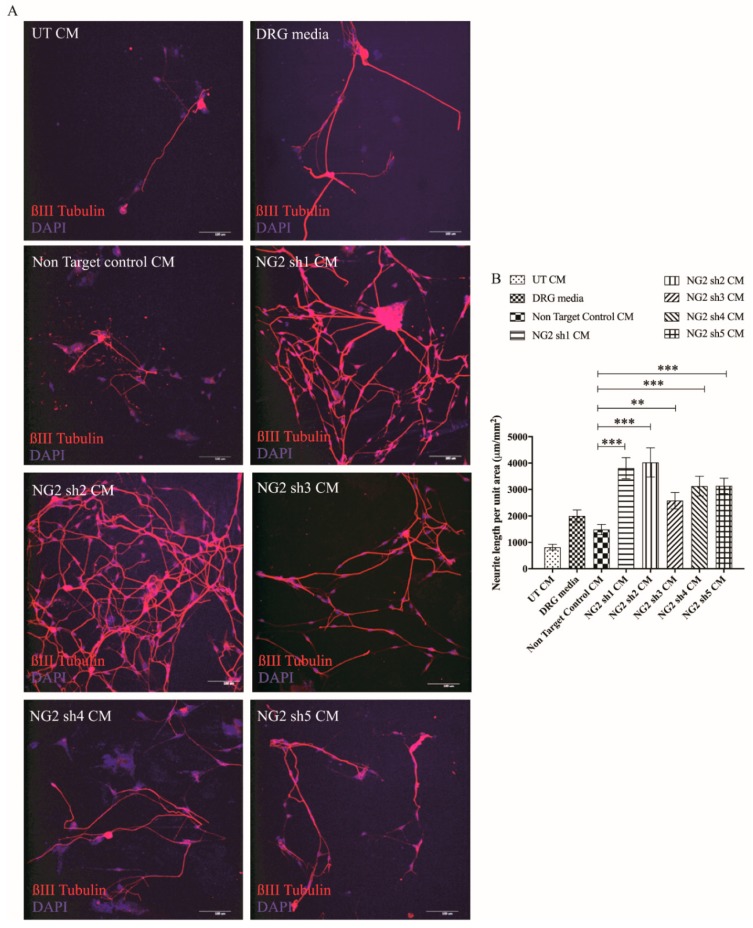
The conditioned medium from shNG2 lentiviral vector transduced Neu7 cells promotes neurite outgrowth in vitro. Photomicrographs show representative projected confocal images of DRG neurons after treatment with conditioned media (CM) from untransduced Neu7 cells (UT), DRG media, non-targeting control, NG2 sh1,NG2 sh2, NG2 sh3, NG2 sh4 and NG2 sh5 (**A**). Scale bar = 100 μm. Graph shows the neurite length per unit area (in μm/mm^2^) measured using stereology compared to non-target control (**B**). Mean ± SEM. * *p* ≤ 0.05, ** *p* ≤ 0.01, *** *p* ≤ 0.001, compared to non-target control. n = 3. Tukey’s post-hoc test using one-way ANOVA.

The astrocytic cell line Neu7 produces high levels of the CSPG NG2, and conditioned medium from these cells inhibits the outgrowth of DRGs [55]. These cells have been used as an in vitro model of neurite outgrowth inhibition such as that caused by the glial scar in SCI. Neu7 inhibition of neurite outgrowth was significantly reduced following transduction with all of shNG2 lentiviral vectors compared to cells transduced with the non-targeting shRNA vector (Figure 5). This was demonstrated by measuring the length of βIII-tubulin-stained DRG neurons in vitro (Figure 5A,B). These results suggest that lentiviral shRNAs against NG2 reduced levels of NG2 protein production by Neu7 cells and that these reduced levels of NG2 were not sufficient to inhibit neurite outgrowth in this in vitro functional assay.

These cell culture experiments demonstrated that transduction with NG2sh1, NG2sh2 and NG2sh5 shRNAs significantly reduced the level of NG2 protein produced by Neu7 cells and also significantly relieved the Neu7 inhibition of DRG cell outgrowth. NG2 sh1 was selected and used for the rest of the studies described here.

### 3.5. Spinal Cord Slice Cultures Transduced with NT-3 Lentiviral Vector Produce NT-3 Protein

**Figure 6:**
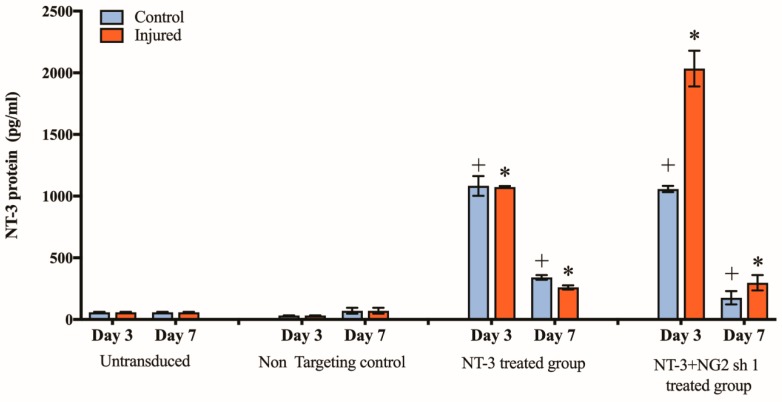
NT-3 protein is released into conditioned media after Lenti-NT-3 transduction of spinal cord slices. Graph shows the NT-3 protein concentration (in pg/mL) measured in the media harvested from control and injured, untransduced and transduced spinal cord slices. The culture media were harvested at days 3 and 7 and untransduced slices were used as a control. Mean ± SEM, + *p* ≤ 0.001 significant increase from day 3 and 7 untransduced control group, * *p* ≤ 0.001 significant increase from day 3 and 7 untransduced injured group. The mean differences were analysed using two-way ANOVA. n = 3 litters (12 pups per litter) which relates to six slices per control group per time-point and nine slices per injured group per time-point for each litter.

To ascertain whether control and injured (transected) spinal cord slices transduced with Lenti-NT-3 vector alone or combination of Lenti-NT-3/NG2 sh1 vectors produce NT-3 protein, the conditioned media were collected 3 and 7 days post-transduction of the slice cultures and tested by ELISA (Figure 6). All of the OSCs transduced with NT-3 or NT-3/NG2 sh1 produced significantly more NT-3 protein than the untransduced OSCs or slices transduced with the non-targeting shRNA control vector (Figure 6). A comparison showed that the uninjured and injured slices produced similar levels of NT-3 after transduction with NT-3, but higher levels of NT-3 were measured in the conditioned media of slices transduced with a combination of Lenti-NT-3/NG2 sh1. The reason for this increased level of NT-3 following co-transduction with Lenti-NG2 sh1 is not yet understood. The levels of NT-3 measured at day 7 were lower than those observed at day 3 for slices transduced with NT-3 or with NT-3/NG2; however, they remained significantly higher than the control slices (Figure 6). Previous studies in our group have shown that there is not a significant decrease in cell viability between day 3 and day 10 post-injury [53]. As lentiviral transduction leads to integration in the host cell, this change is not because of the vector (unlike transient changes following transduction with other vectors such as adenoviral vectors), but this decrease may reflect changes in protein production by cells during the slice culture).

### 3.6. NG2 Expression Is Reduced in Ex Vivo Spinal Cord Slice Cultures 7 Days after Transduction with Lenti-NT-3/NG2 sh1

Having validated the functions of the Lenti-NT-3 and Lenti-NG2 sh1 vectors in vitro, the aim of this portion of the study was to determine whether these vectors altered the injury environment of an ex vivo OSC model of the injured spinal cord. We developed an ex vivo transection model of SCI and used stereology to show that the volume fraction (Vv) of NG2 proteoglycans increased significantly between day 3 and day 10 post-injury [5]. The OSC was analysed as 3 separate zones, the injury zone (IZ) i.e., the lesion site, the scar zone (SZ) defined as a distance of 100 μm from the edge of the lesion site and the near scar zone (NSZ) defined as a 100 μm distance from the SZ. Control or injured OSCs were cultured for 3 days, viral vector was added then the slices were maintained in culture for 7 days prior to analysis by immunostaining.

**Figure 7:**
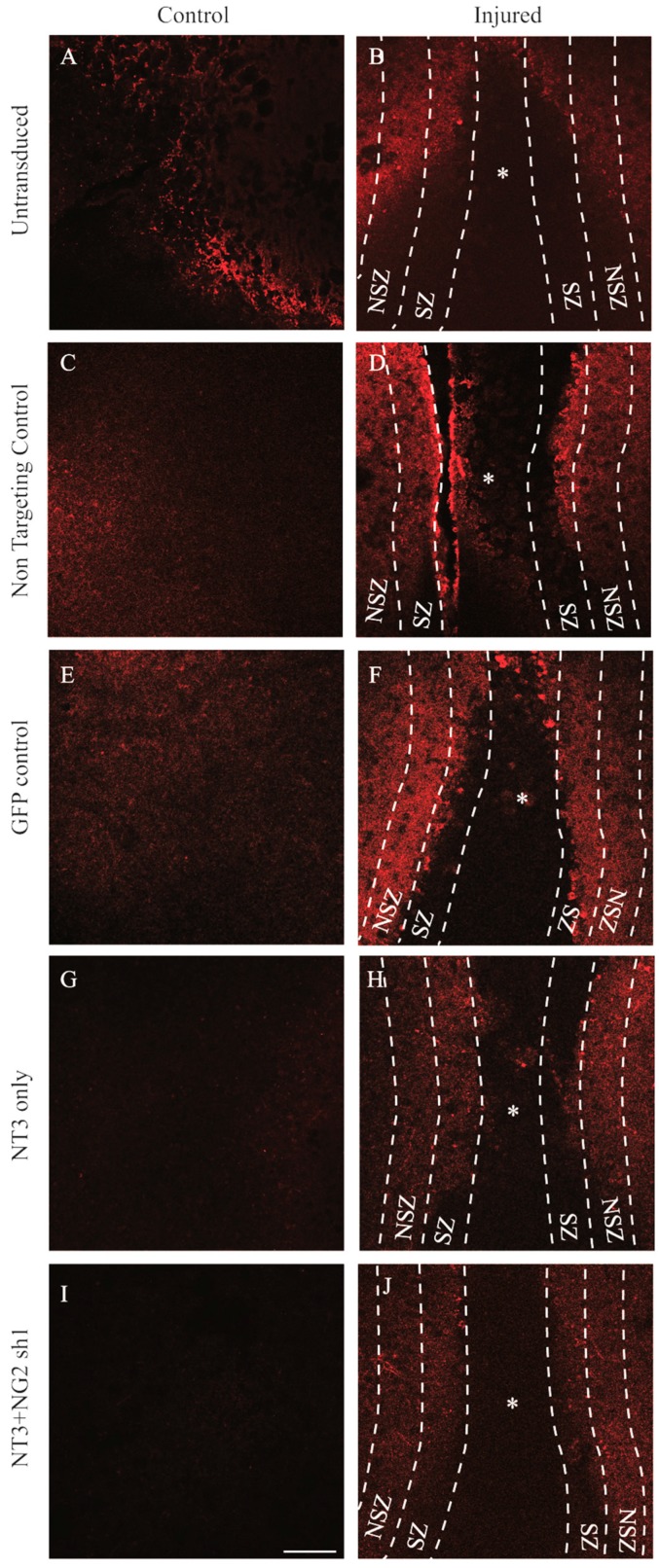
Confocal images showing NG2 levels in untransduced and lentiviral vector transduced control and transection injured ex vivo spinal cord slices. Photomicrographs show projected confocal images of NG2 (red) in untransduced (**A**,**B**), non-targeting control (**C**,**D**), Lenti GFP transduced (**E**,**F**), Lenti-NT-3-transduced (**G**,**H**) and Lenti-NT-3/NG2 sh1 transduced (**I**,**J**) control and injured slices, respectively. * = injury zone (IZ); dashed lines separate the regions of interest examined: scar zone (SZ) and near scar zone (NSZ). SZ and NSZ were 100 μm in width. Scale bar = 100 μm.

**Figure 8:**
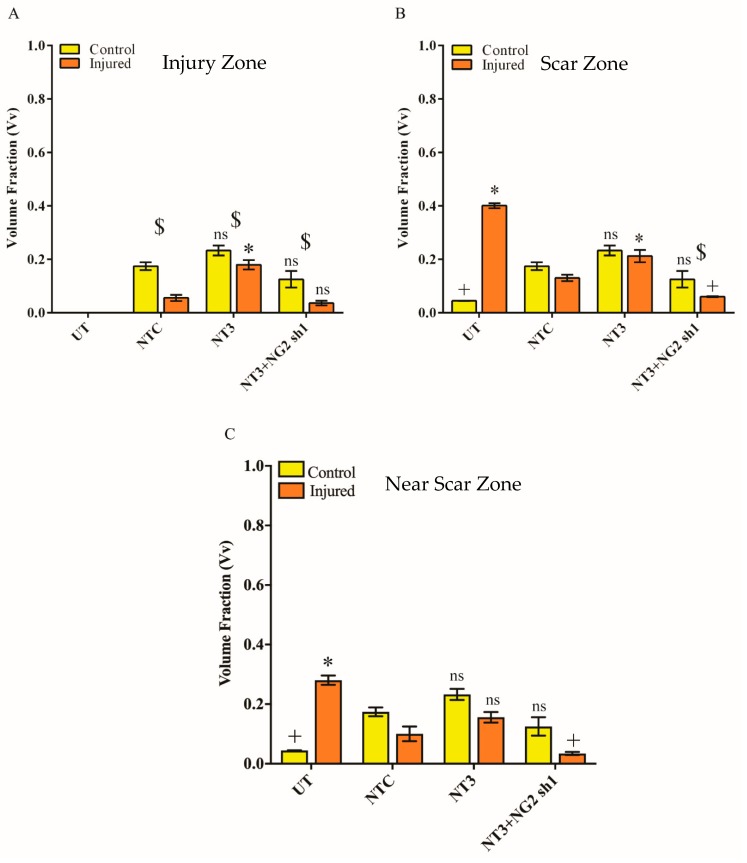
Vv of NG2 in untransduced and lentiviral vector transduced control and transection injured ex vivo spinal cord slices. Graphs show Vv NG2 immunohistochemical staining at the IZ (**A**), SZ (**B**) and NSZ (**C**). UT = untransduced, NTC = non-targeting control. Mean ± SEM. * *p* ≤ 0.001 significant increase from NTC, + *p* ≤ 0.001 significant decrease from NTC, $ *p* ≤ 0.001 significant difference between control and injured groups, ns indicates no significant difference compared to the NTC or between control and injured groups. The mean differences were analysed using two-way ANOVA. n = 3 litters (12 pups per litter) which relates to six slices per control group per time-point and nine slices per injured.

NG2 levels were quantified by immunostaining and stereological analysis to determine the Vv of NG2 staining. As expected, after injury to the slice and in the absence of transduction, the Vv NG2 increased in the regions adjacent to the injury (SZ and NSZ) (Figure 7 and Figure 8B). Following transduction with Lenti-NT-3/NG2 Sh1, the SZ and NSZ regions of transected OSCs showed significantly lower levels of NG2 staining than slices transduced with the non-targeting shRNA.

The analysis shows an interesting and as yet unexplained observation in the SZ and NSZ regions of injured slices where transduction with lentiviral non-targeting control shRNA caused a significant reduction in NG2 compared to untransduced slices. In addition, there appeared to be a significant increase in the Vv of NG2 in control and injured slices following Lenti-NT-3 transduction, but this was significantly reduced by transduction with the Lenti-NT-3/NG2 sh1 vector combination (Figure 8). This may be because NT-3 stimulates the production or proliferation of NG2-positive early precursor cells [57]

3.7. βIII-Tubulin Levels Are Increased in Ex Vivo Spinal Cord Slice Cultures Transduced with Lenti-NT-3/NG2 sh1

**Figure 9:**
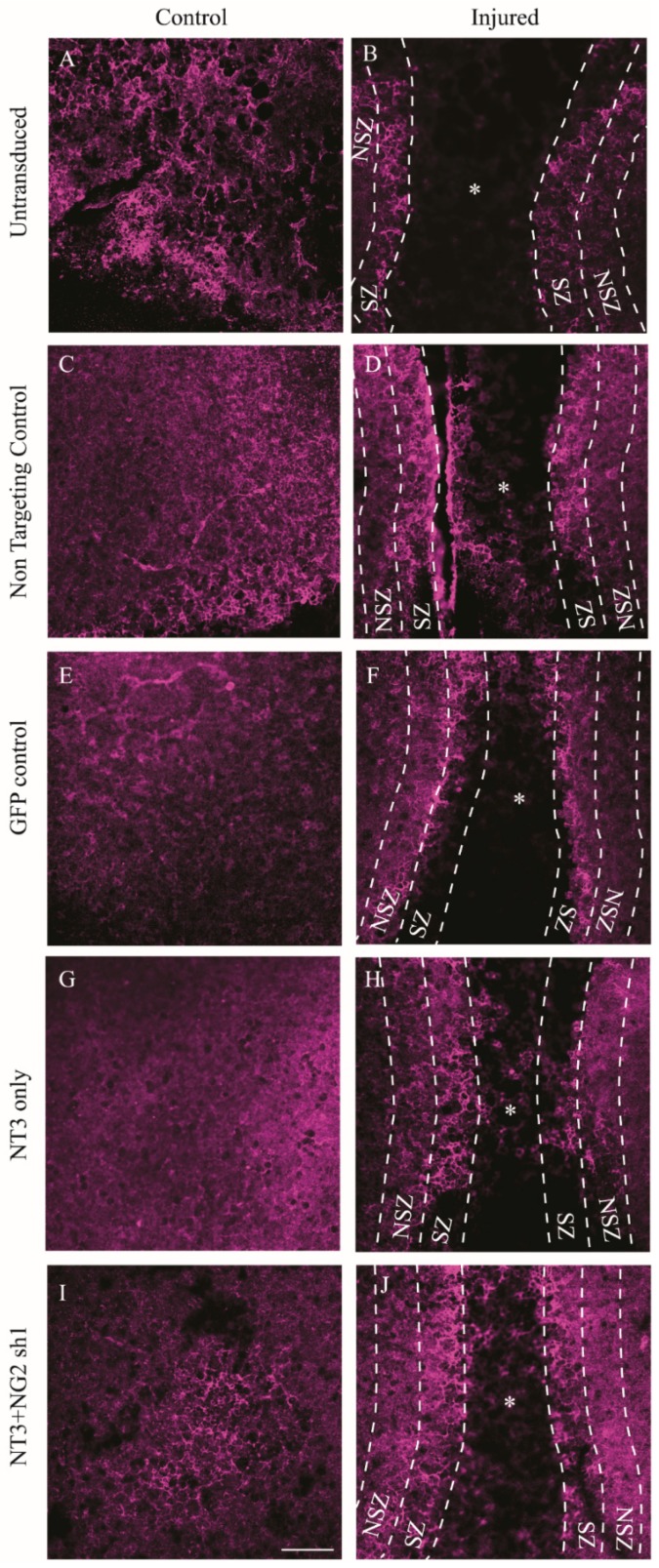
Confocal images showing βIII-tubulin expression in untransduced and lentiviral vector transduced control and transection injured ex vivo spinal cord slices. Photomicrographs show projected confocal images of βIII-tubulin (purple) in untransduced (**A**,**B**), non-targeting control (**C**,**D**), Lenti GFP transduced (**E**,**F**), Lenti-NT-3-transduced (**G**,**H**) and Lenti-NT-3/NG2 sh1 transduced (**I**,**J**) control and injured slices, respectively. * = injury zone (IZ). Dashed lines separate the regions of interest examined; SZ and NSZ were 100 μm in width. Scale bar = 100 μm.

**Figure 10:**
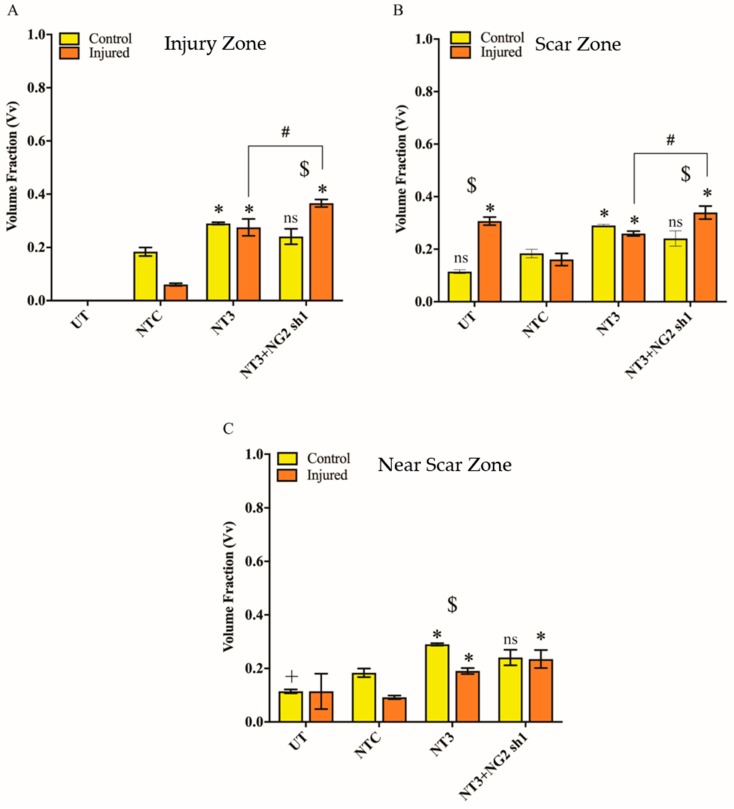
Vv βIII-tubulin in untransduced and lentiviral vector transduced control and transection injured ex vivo spinal cord slices. Graphs show Vv βIII-tubulin immunohistochemical staining at the IZ (**A**), SZ (**B**) and NSZ (**C**). UT = untransduced, NTC= non-targeting control. Mean ± SEM. * *p* ≤ 0.001 significant increase from NTC, $ *p* ≤ 0.001 significant difference between control and injured slices in each group, # *p* ≤ 0.01 significant difference between NT-3 and NT-3/NG2 sh1. ns indicates no significant difference compared to the NTC or between control and injured groups. The mean differences were analysed using two-way ANOVA. n = 3 litters (12 pups per litter) which relates to six slices per control group per time-point and nine slices per injured group per time-point for each litter.

The Vv of βIII-tubulin staining in each region of the OSCs was assessed as a measure of the proportion of the OSC volume occupied by neurons. Within the IZ, as expected, injury led to a decrease in Vv βIII-tubulin compared to uninjured samples. Transduction with Lenti-NT-3 significantly increased the Vv βIII-tubulin compared to samples treated with a control vector suggesting a stimulation of axonal growth caused by the increase in NT-3 (Figure 9 and Figure 10A). The Vv βIII-tubulin was further significantly increased by transduction with a combination of Lenti-NT-3/NG2 sh1 compared to Lenti-NT-3 alone suggesting that a reduction in NG2 enhances axonal sprouting/growth. Likewise within the SZ a significant increase in the Vv βIII-tubulin was observed for OSCs transduced with Lenti-NT-3 vs. those transduced with a control vector. This increase was observed in both control and injured OSCs. Similar to results observed in the IZ, within the SZ, the Vv βIII-tubulin was further increased by transduction with the Lenti-NT-3/NG2 sh1 combination compared to Lenti-NT-3 alone, but only for injured OSCs (Figure 9 and Figure 10B). This suggests that a within the IZ and SZ, stimulation of axonal growth is enhanced by increased NT-3 and further enhanced by a reduction in NG2 (Figure 9 and Figure 10). Unexpectedly, the Vv βIII-tubulin in the SZ increased following injury in untransduced OSCs even though the Vv NG2 increased in the same samples.

Further away from the site of injury, in the NSZ Lenti-NT-3 also resulted in an increase in Vv βIII-tubulin compared to the control transduction suggesting enhanced axonal growth (Figure 9 and Figure 10C). However, unlike the IZ and SZ, transduction with a combination of Lenti-NT-3 and NG2 sh1 did not lead to a statistically significant increase in Vv of βIII-tubulin compared to Lenti-NT-3 alone in either control or injured OSCs. This suggests that at some distance from the glial scar, where NG2 is not as abundant, knockdown of NG2 does not enhance axonal outgrowth (Figure 7, Figure 8, Figure 9 and Figure 10). Thus, the greatest increase in Vv βIII-tubulin-positive staining in injured OSCs was observed following delivery of a combination of NT-3 and NG2 sh1 vectors.

## 4. Discussion

Ex vivo models of SCI allow researchers to mimic in vivo injury and to optimise novel interventions before moving into preclinical studies supporting the three Rs (replacement, reduction, refinement) of animal usage. Here we describe an ex vivo rat model of spinal cord transection injury for testing a lentiviral vector-based treatment to overcome the inhibitory environment for regeneration at the lesion site. To our knowledge this is the first report testing a lentiviral vector in an organotypic slice culture model of SCI.

Neurotrophic factors are widely recognised as potential therapeutic factors in the treatment of SCI [58,59]. Many studies have shown that NT-3 can increase neurite outgrowth and improve functional outcomes in vivo. We show here that transduction with the NT-3 lentiviral vector leads to a significant increase in NT-3 protein production in vitro in cultured cells (Figure 1) and ex vivo in organotypic spinal cord slice cultures (Figure 6). The NT-3 produced by transduced cells was able to promote an increase in DRG neurite length compared to control cell medium (Figure 2). These findings are consistent with work by Donnelly et al. [36] who showed that Lenti-NT-3-transduced cells secreted NT-3 at sufficient levels (>100 pg/mL) to promote neurite outgrowth [36].

NG2 is regarded as one of the key CSPGs that causes axonal growth inhibition at the lesion site following an SCI [40,42]. Gene therapy approaches whereby an inhibitory gene is knocked down using shRNA have proved a promising approach in the potential treatment of SCI [60,61]. Here, Lenti-NG2 sh1 knockdown of the rat NG2 protein was validated in Neu7 cells (Figure 3 and Figure 4). This NG2 knockdown was sufficient to relieve the inhibition of DRG neurite outgrowth caused by Neu7 conditioned medium (Figure 5). We demonstrate here that the combination of Lenti-NT-3/NG2 sh1 causes significant reduction of NG2 protein in ex vivo spinal cord slice cultures compared to the non-targeting control group (Figure 7 and Figure 8). This is consistent with the observations in an in vivo contusion model of SCI where rats were treated with combination Lenti-NT-3/NG2 shRNA [35]. This suggests that the organotypic slice culture model represents a potentially meaningful alternative to in vivo SCI models for studying strategies to target the glial scar.

We examined βIII-tubulin expression as a marker of neurons in the IZ, SZ and NSZ. The Vv βIII-tubulin was significantly higher in injured spinal cord slice cultures transduced with combination of Lenti-NT-3/NG2 sh1 than those treated with the non-targeting control in all zones examined (Figure 9 and Figure 10). These results suggest that a combination of Lenti-NT-3 and Lenti-NG2 sh1 promote a positive microenvironment for axonal regrowth by reducing NG2 expression and increasing NT-3 levels.

In addition, our results also showed significant βIII-tubulin expression following transduction with Lenti-NT-3 alone compared to non-targeting control (even without a reduction in NG2 protein), in particular in the IZ, suggesting that the increase in axonal growth, is localised and appeared to be short distance sprouting at the lesion site compared to Lenti-NT-3/NG2 sh1 transduced slices (Figure 9). This result is in agreement with Taylor et al., who suggest that the growth of axons after Lenti-NT-3 transduction is short distance growth and the neurotrophic stimulus alone is not enough to stimulate long distance axonal outgrowth and sprouting [20].

We have shown using cultured cells and organotypic spinal cord slice cultures that the NT-3 coding sequence and shRNA sequences against NG2 protein can be efficiently delivered using lentiviral vectors. The combination of neurotrophic support (via Lenti-NT-3) and downregulation of NG2 (via Lenti-NG2 sh1) provides a positive microenvironment for axonal growth and less inhibitory glial scar activity around the injury site in this ex vivo model of SCI. The results obtained using this ex vivo model of injury are consistent with in vivo studies suggesting that this ex vivo injury model may be a valid one for assessing the initial feasibility of strategies to enhance neuronal regeneration in a manner consistent with the three Rs.

However, the ex vivo OSC has a number of limitations which means it cannot replace all in vivo studies. Firstly, because the slice culture system lacks a blood supply throughout the study, the effect of blood circulating immune cells (e.g., monocytes and macrophages) which infiltrate the damaged tissue and respond to cytokines post-injury cannot be ascertained using this ex vivo system. Tissue resident microglia/macrophages do accumulate at the edges of slices due to damaged caused by the cutting and slicing procedures and in injured regions. Secondly, this study used slices derived from newborn (P4) pup spinal cords as these reportedly have better neuronal survival and a more consistent longitudinal fibre tract development than slice culture from animals beyond P6 [62]. Slice cultures from adolescent/adult animals (8 weeks and over) have been reported, but these were observed to have decreased vitality and tissue organisation compared to neonatal slices [8] OSC models based on neonatal spinal cords may not reflect the known differences between SCI responses in neonatal and adult animals; for example, studies in vivo demonstrated a more intense and extensive astrogliosis in adults following SCI than in neonatal animals [63]. There may be differences in the susceptibility of cells to be transduced with viral vectors between the developing and the adult spinal cord. Ideally this study would be repeated with slices derived from the spinal cords of mature rats in order to compare the way the vectors function in neonatal and adult rats. Thirdly, this model was used to assess relatively short term changes in the spinal cord post-injury (7 days), whereas SCI in humans is a complex and dynamic environment in which changes occur over much longer periods of time. Finally, perhaps the most significant drawback of using this system is that functional and behavioural assessment cannot be conducted to evaluate a new therapy, nor can complications such as allodynia be measured. Thus, although the model is useful for initial evaluations of new therapies, in vivo testing for efficacy and safety is still required.

## Figures and Tables

**Table 1:** Summary of sequence alignment of shRNA targeting NG2 between *Rattus norvegicus* and *Mus musculus*. The mismatched base pairs between two species are shown in cyan as shown in NG2 sh3, sh4 and sh5. Key: sh: short hairpin RNA; CSPG4: chondroitin sulphate proteoglycans 4.

ShRNAs	Species	DNA Sequences Targeting CSPG4 (NG2)	Alignment
NG2 sh1	*Rattus norvegicus*	…GGGACAAGCGTGGCAACTTTATCTA…	Match
Catalogue number TRCN0000348358	*Mus musculus*	…GGGACAAGCGTGGCAACTTTATCTA…	
NG2 sh2	*Rattus norvegicus*	…GCATAGAAGATTTCAGTGTTAATGG…	Match
Catalogue number TRCN0000098337	*Mus musculus*	…GCATAGAAGATTTCAGTGTTAATGG…	
NG2 sh3	*Rattus norvegicus*	…GGGTATCTCCACGTAGCCAATAGT…	One base mismatch
Catalogue number TRCN0000334788	*Mus musculus*	…CCCTATCTTCACGTAGCCAATAGT…	
NG2 sh4	*Rattus norvegicus*	…CAATACCCTACACGTACTTTCAACC…	One base mismatch
Catalogue number TRCN0000348354	*Mus musculus*	…CAATACCCTACGCGTACTTTCAACC…	
NG2 sh5	*Rattus norvegicus*	…GCAACCAACTTGTGGAAGATTTC…	One base mismatch
Catalogue number TRCN0000098338	*Mus musculus*	…GCAACCAACTTGTGGAACATTTC…	

## Supplementary Materials

The following are available online at https://www.mdpi.com/2079-7737/9/3/54/s1, Figure S1: Schematic diagram of the shNG2, TurboGFP^TM^ and pWPT-GFP vectors. Figure S2: Immunoblot of NG2 and Actin showing full blot and molecular weight markers. Table S1: Relative NG2/ β-actin intensities from 3 separate immunoblots. Figure S3: NG2 sh1 lentiviral vector significantly reduces NG2 protein in Neu7 cells in vitro.
