# Supplementary material for: Ex Vivo Rat Transected Spinal Cord Slices as a Model to Assess Lentiviral Vector Delivery of Neurotrophin-3 and Short Hairpin RNA against NG2
Source: Biology (Basel). 2020 Mar 15;9(3):54. doi: 10.3390/biology9030054 (PMC7150802; doi:10.3390/biology9030054)
Supplement: Supplementary file 1 [file biology-09-00054-s001.pdf]

Supplementary Materials

Ex Vivo Rat Transected Spinal Cord Slices as a Model to Assess Lentiviral Vector Delivery of Neurotrophin-3 and Short Hairpin RNA against NG2

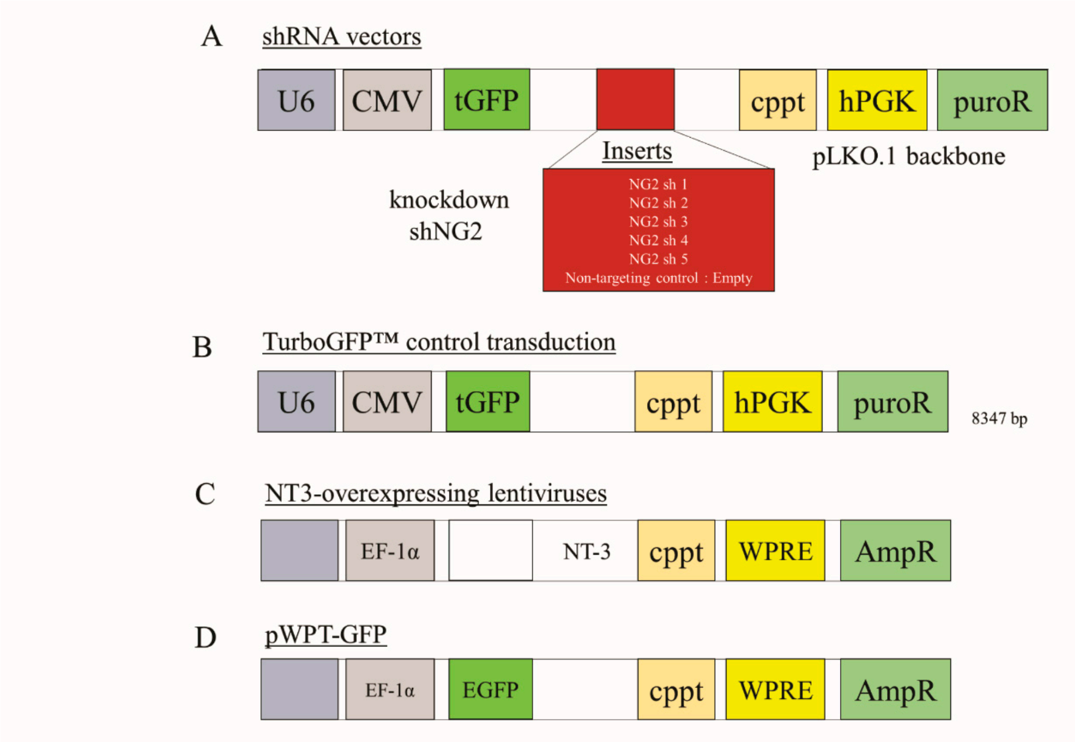

Figure S1. *Schematic diagram of the shNG2, TurboGFP™ and pWPT-GFP vectors.* Puromycin resistance gene (puroR) provides a selectable marker for transduction by the shNG2 vector (A). Non-Targeting Control vector contains a shRNA insert that does not target any known genes from any mammalian species and acts as negative control (A). TurboGFP™ control vector contained a sequence encoding TurboGFP™, under the control of CMV promoter (B). NT-3 expressing vector contained a sequence encoding NT-3 gene under the control of EF-1α promoter (C). pWPT-GFP control vector containing GFP sequence under the control of EF-1α promoter (D). Key: U6, U6 promoter; hPGK, human Phosphoglycerate Kinase eukaryotic promoter; CMV, Cytomegalovirus promoter; puroR, puromycin resistance gene; tGFP, turbo green fluorescent protein; AmpR, Ampicilin resistance gene; cppt, central polypurine tract; WPRE, Woodchuck Posttranscriptional Regulatory Element; EF 1-α,human Elongation Factor 1-alpha.

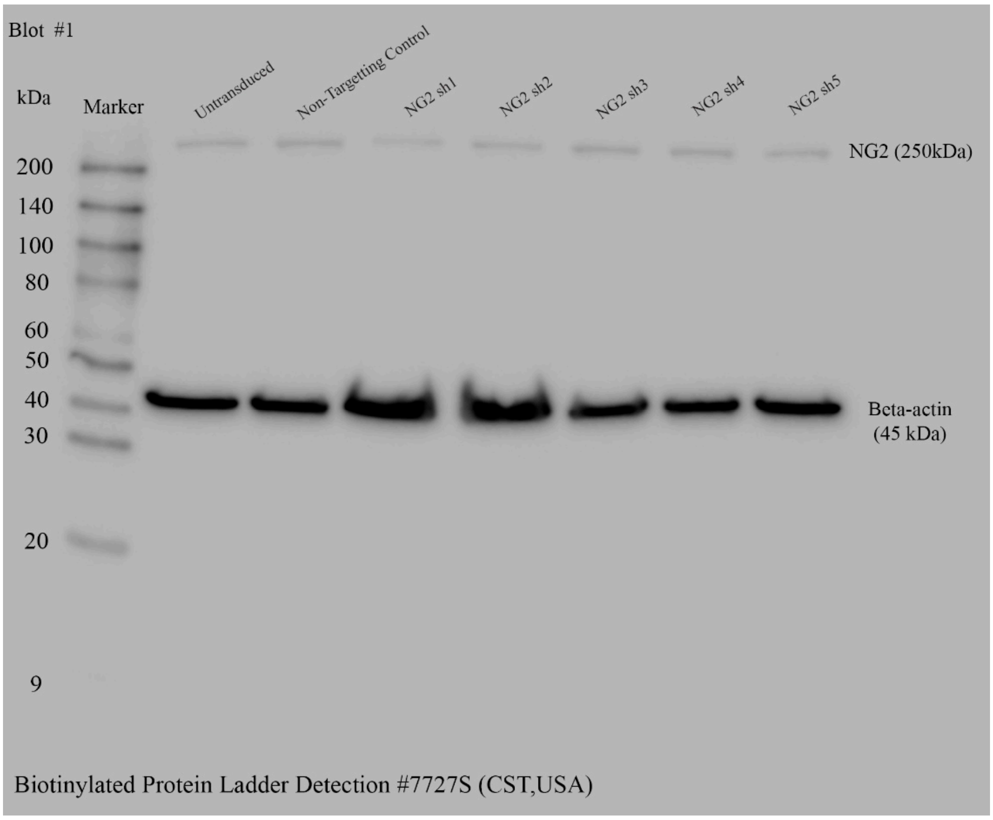

Figure S2. *Immunoblot of NG2 and Actin showing full blot and molecular weight markers.*

Table S1. Relative NG2/ β-actin intensities from 3 separate immunoblots.

	Relative NG2/ β-Actin signal				
**Repeat #**	**1**	**2**	**3**	**Mean**	**SD**
UT	0.025415	0.027450	0.029557	0.027474	0.002071
NTC	0.024632	0.027911	0.027850	0.026798	0.001876
NG2 sh1	0.001267	0.001347	0.001284	0.001299	0.000042
NG2 sh2	0.003741	0.002997	0.003647	0.003462	0.000405
NG2 sh3	0.020684	0.021470	0.022440	0.021531	0.000880
NG2 sh4	0.038260	0.031220	0.035337	0.034939	0.003537
NG2 sh5	0.010677	0.015220	0.017080	0.014326	0.003294

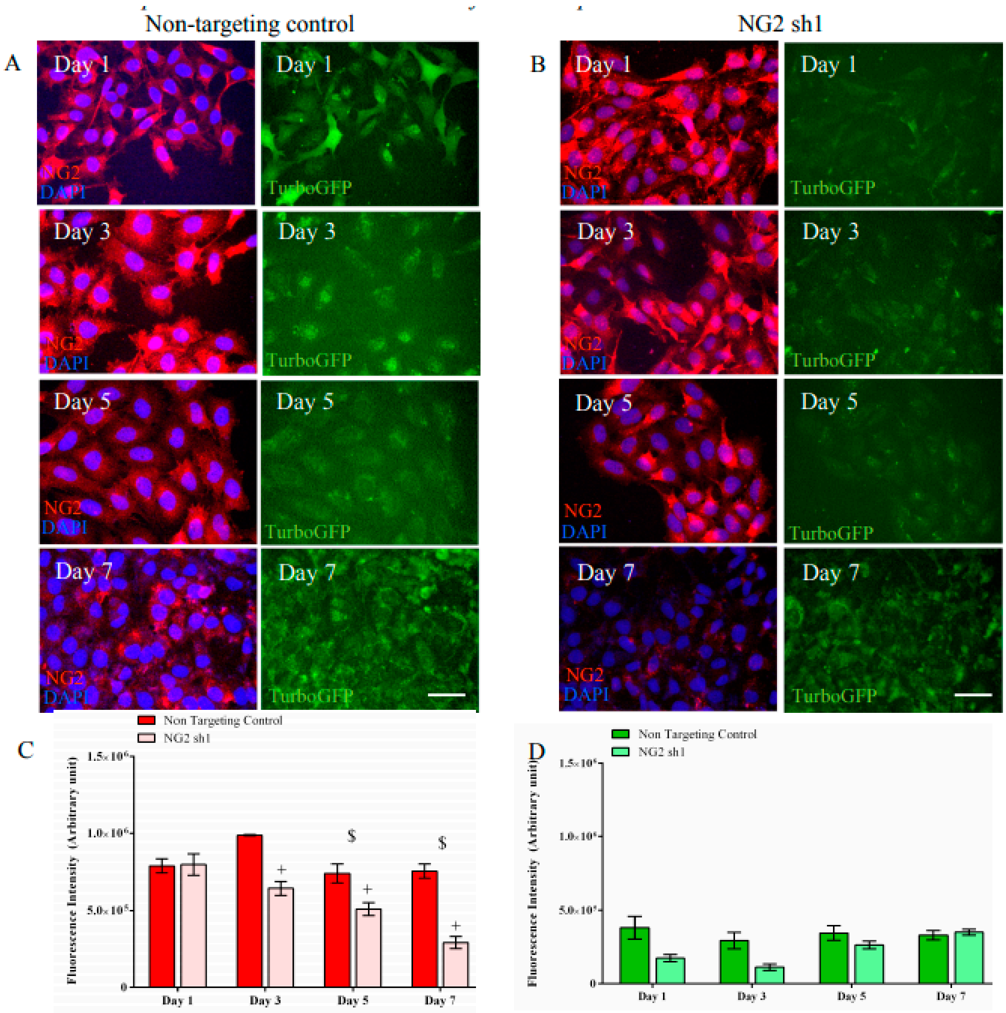

Figure S3. *NG2 sh1 lentiviral vector significantly reduces NG2 protein in Neu7 cells in vitro.* Photomicrographs show representative projected/stacked confocal images of corrected total fluorescence of NG2 protein on days 1, 3, 5 and 7 post transduction with non-targeting control (A) or NG2 sh1 lentiviral vectors (B). Scale bar=50 µm. Graphs shows the corrected total cell fluorescence of NG2 protein at each time point (C) and the total cell fluorescence of GFP+ in Neu7 cells (D). Mean ± SEM, + $p \leq 0.001$ significant decrease compared to Non Targeting Control (NTC) group, \$$p \leq 0.001$ significant compared to days. Analysis was performed using Two-way ANOVA with Tukey's multiple comparisons test. N=3 which relates to 4 independent wells of NTC and of NG2 sh1 treated group per time-point for each technical repeat.

© 2020 by the authors. Submitted for possible open access publication under the terms and conditions of the Creative Commons Attribution (CC BY) license (http://creativecommons.org/licenses/by/4.0/).
